# Supplementary figures and images for: Elucidating the Mechanism by Which HIV-1 Nucleocapsid Mutations Confer Resistance to Integrase Strand Transfer Inhibitors
Source: bioRxiv. 2025 May 18:2025.05.17.654662. Preprint. [Version 1] doi: 10.1101/2025.05.17.654662 (PMC12478370; doi:10.1101/2025.05.17.654662)

Extended data 1

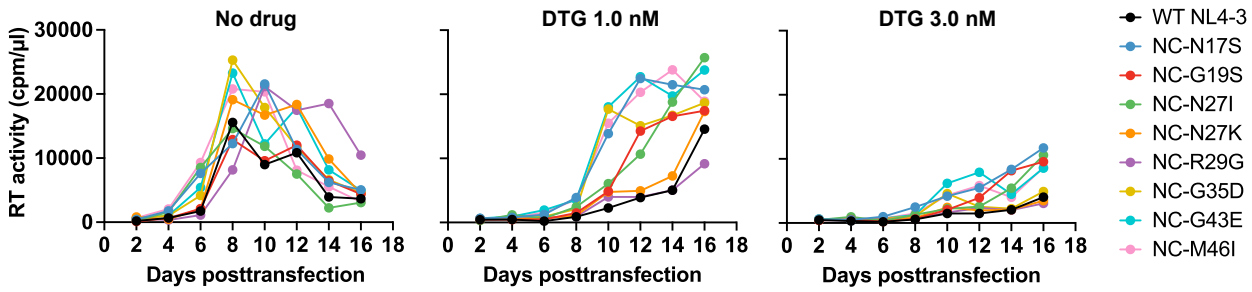

Supplement: Supplement 1 — Extended data 1. Replication kinetics of the NC mutants selected with DTG in the SupT1 T-cell line. Replication kinetics of the indicated NL4–3 variants in the SupT1 T cell line in the absence or presence of DTG. Replication curves obtained in the presence of 0, 1, and 3 nM DTG are shown. Data are representative of three independent experiments. [file media-1.pdf]

Extended data 2

A

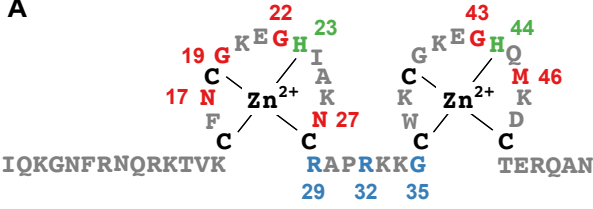

B

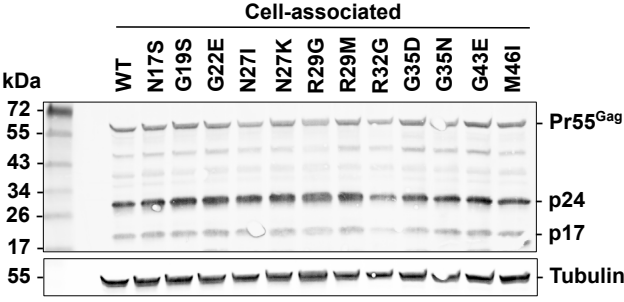

C

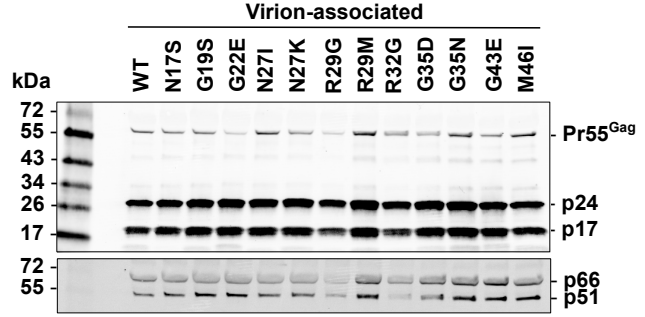

D

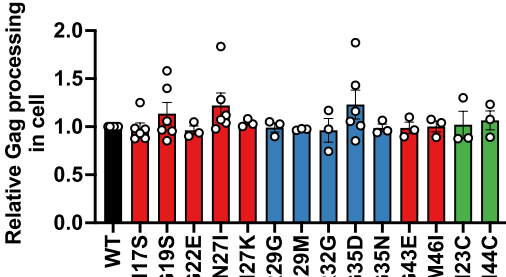

E

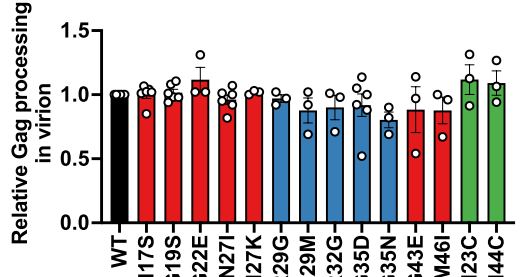

F

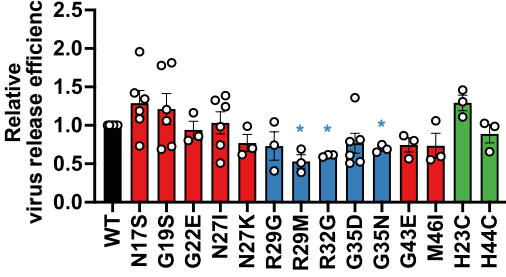

G

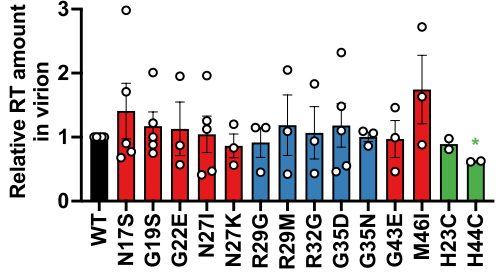

H

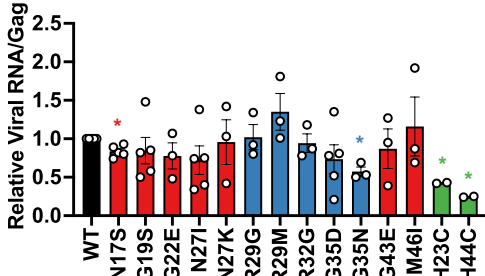

Supplement: Supplement 2 — Extended data 2. NC mutations selected in the presence of INSTIs do not affect virion composition or viral release efficiency. (A) The NC mutations investigated in this study. Mutated residues in the zinc-finger domain and basic linker are highlighted as red and blue, respectively. Control mutations in the CCHC motif are shown in green. Western blot analysis of the virus-producing 293T cells (B) and virion fraction produced from these cells (C), probed with anti-Gag Ab, anti-tubulin Ab, or anti-RT Ab. Representative western blots from at least three independent experiments are shown. Quantification of Gag processing in the virus-producing cells (D) and virions (E). Gag processing was expressed as the ratio of p24 compared to total p24 and Pr55Gag. (F) Virus release efficiency was expressed as the ratio of virion p24 compared to the total virion p24, cell p24, and cell Pr55Gag. The data are shown as means ± SEM from at least three independent experiments (G) Quantification of RT enzyme in the virion. The relative ratio of total RT (p66 and p51) per virion p24 is shown. The data are shown as means ± SEM from at least two independent experiments with statistical significance indicated (*p < 0.05) as per one sample t-test. (H) Viral RNA packaging in the virion. Viral RNA was extracted from virions and subjected to qRT-PCR. The viral RNA values were normalized by the quantification of total Gag bands (p24 and Pr55Gag) in the western blots shown in panel C. The data are shown as means ± SEM from at least two independent experiments with statistical significance indicated (*p < 0.05) as per one-sample t-test. [file media-2.pdf]

Extended data 4

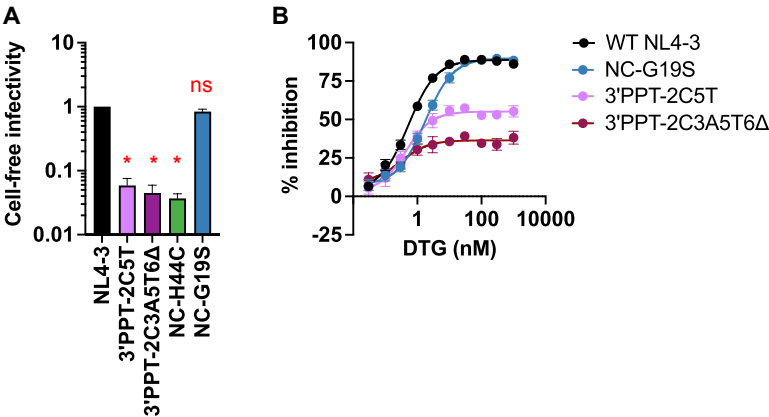

Supplement: Supplement 4 — Extended data 4. Cell-free infectivity and DTG sensitivity of NC-H44C and 3’PPT mutants. (A) Cell-free infectivity of WT NL4–3, 3’PPT mutants, NC-H44C, and NC-G19S in TZM-bl cell. Relative infectivity is shown, normalized to 1 for WT NL4–3. The data are shown as means ± SEM from at least two independent experiments with statistical significance indicated (*p < 0.05) as per one sample t-test. (B) DTG sensitivity of the NC-G19S and 3’PPT mutants. TZM-bl cells were incubated with TCID50-normalized WT virus or the indicated mutants in the presence of a range of DTG concentrations. The data are shown as means ± SEM from >3 independent experiments. [file media-4.pdf]

Extended data 5

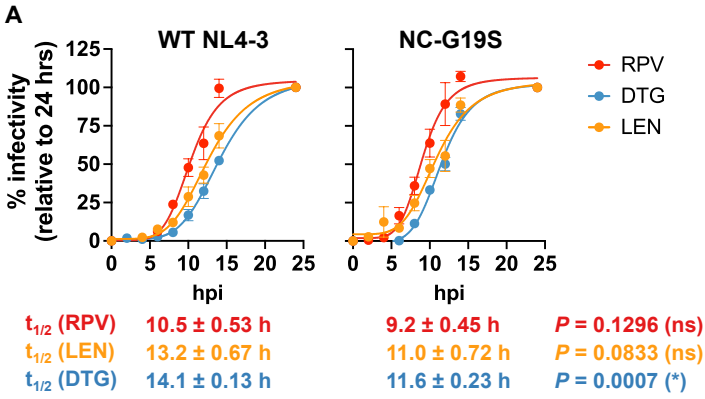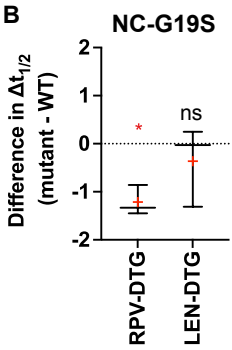

Supplement: Supplement 5 — Extended data 5. Time-of-addition assay in HeLa cells using an HIV-1 reporter virus encoding mScarlet. (A) HeLa cells were infected with VSV-G-pseudotyped mScarlet reporter virus, followed by the addition of RPV, LEN and DTG at the indicated time points. mScarlet expression at the indicated time points was normalized to the signals at 24 h post-infection. t1/2 for the indicated drugs is shown below the graph. The data are shown as means ± SEM from >3 independent experiments. (B) Differences in Δt1/2 (the difference in t1/2 between DTG and other drugs) between WT and the NC-G19S mutant.. Box plots show median, interquartile range, minimum, and maximum values. The mean value is indicated by a ‘+’. Statistical significance is shown (*p < 0.05) as determined by a one-sample t-test, ns: not significant. [file media-5.pdf]
