# Supplementary material for: Elucidating the Mechanism by Which HIV-1 Nucleocapsid Mutations Confer Resistance to Integrase Strand Transfer Inhibitors
Source: bioRxiv. 2025 May 18:2025.05.17.654662. Preprint. [Version 1] doi: 10.1101/2025.05.17.654662 (PMC12478370; doi:10.1101/2025.05.17.654662)
Supplement: Supplement 3 — Extended data 3. Analysis of 2-LTR circle junctions in total DNA isolated from infected SupT1 T-cells at 24 h post-infection. PCR-amplified 2-LTR junctions were cloned into the pCR-Blunt II-TOPO vector for sequencing. (A) Representative examples of 2-LTR junctions. Junctions were categorized as having intact (GTAC) sequences, deletions (≥3 nucleotides), insertions, or small substitutions at the GTAC motif. “X” denotes any nucleotide, while “*” indicates deletions. Notably, extended U3 sequences with 3′PPT retention were observed exclusively in 2-LTR junctions of the 3′PPT-2C3A5T6Δ mutant. (B) The total number of sequences analyzed for each variant is indicated. n indicates the number of experimental repeats. (C) Statistical analysis of trends in 2-LTR modification. The chi-squared test was used to compare overall and individual trends in modified 2-LTR junction frequencies. The categories with significant differences were highlighted in light blue (*p < 0.05). ns: not significant. (E) Integration site frequency in cells infected with WT or NC-G19S. IS: integration site, RefSeq genes: genes annotated in the NCBI Reference Sequence Database, TSS: transcription start sites of RefSeq genes, SPAD: speckle-associated domains, LAD: lamina-associated domains. The binomial test was used to compare WT and NC-G19S (*p < 0.05). ns: not significant. (F) The frequency of the integration events with canonical and aberrant viral DNA ends at 5’LTR and 3’LTR. The binomial test was used to compare WT and NC-G19S (*p < 0.05). ns: not significant. [file media-3.pdf]

Extended data 3

A

U5	U3	
AGTGAATTAGCCCTTCCAGT	ACGCTAGAGATTTTCCAC	Consensus GTAC
AGTGAATTAGCCCTTCCAXX--insert--XXGCTAGAGATTTTCCAC		Insertion
AGTGAA*****del******--insert--*****del*****TCCAC		Insertion
AGTGAA*****del******	****del****TTTCCAC	Deletion
AGTGAA*****del******	ACGCTAGAGATTTTCCAC	Deletion
AGTGAATTAGCCCTTCCAGT	****del****TTTCCAC	Deletion
AGTGAATTAGCCCTTCCAXX	XXGCTAGAGATTTTCCAC	Substitution
AGTGAATTAGCCCTTCCAGT	ACTGCTTTTCTTTTAAAA	Extended U3 with 3'PPT

3'PPT retention

B

	No. clones (n)
WT NL4-3	433 (5)
NC-G19S	376 (4)
NC-N17S	184 (2)
NC-N27I	177 (2)
NC-H44C	133 (2)
IN-D116N	284 (3)
NC-G19S/IN-D116N	287 (3)
NC-N17S/IN-D116N	193 (2)
NC-N27I/IN-D116N	189 (2)
NC-H44C/IN-D116N	174 (2)
3'PPT-2C3A5T6Δ	92 (1)

C

		P value					
		Overall	Consensus	Insertion	Deletion	Substitution	Extended U3
IN-WT	NC-G19S	0.193	0.710	0.069	0.358	0.797	N.D.
	NC-N17S	0.145	0.155	0.842	0.145	0.750	N.D.
	NC-N27I	0.169	0.276	0.803	0.093	1.000	N.D.
	NC-H44C	< 0.01	0.047	< 0.01	0.734	0.012	N.D.
	3'PPT-2C3A5T6Δ	< 0.01	0.023	< 0.01	0.126	0.418	< 0.01
IN-D116N	NC-G19S/IN-D116N	0.705	0.393	0.633	1.000	0.746	N.D.
	NC-N17S/IN-D116N	0.181	0.087	0.086	1.000	1.000	N.D.
	NC-N27I/IN-D116N	0.226	0.087	0.268	1.000	0.549	N.D.
	NC-H44C/IN-D116N	< 0.01	< 0.01	0.256	< 0.01	0.949	N.D.
	3'PPT-2C3A5T6Δ	< 0.01	< 0.01	0.086	< 0.01	0.478	< 0.01

D

		WT	G19S	Binominal test *P* value
Total unique IS		46,758	44,265	
In RefSeq genes (%)		72.35	71.85	0.0095
In CpG islands (%)	+/- 1kb	0.601	0.549	0.0059
	+/- 2kb	1.722	1.450	< 0.001
	+/- 5kb	5.355	4.810	< 0.001
In TSS of RefSeq genes (%)	+/- 1kb	0.768	0.834	0.0827
	+/- 2kb	2.318	2.465	0.0268
	+/- 5kb	10.231	9.768	<0.001
Median gene density in 1Mb region around IS		13	11	0.4270
In LADs (%)		11.8	13.3	< 0.001
In SPADs (%)		23.5	20.2	< 0.001

E

	WT	G19S	Binominal test *P* value
Total counts	50,366	48,079	
Canonical ends	46,758	44,265	
Aberrant ends	3,608	3,814	< 0.001
3'LTR	3,382	3,541	< 0.001
Deletions	2,596	2,691	< 0.001
Insertions	786	850	< 0.001
5'LTR	226	273	< 0.001
Deletions	1	3	0.0721
Insertions	225	270	< 0.001
% canonical ends	92.84	92.07	
% aberrant ends	7.16	7.93	
3'LTR	6.71	7.36	
5'LTR	0.45	0.57	
